# Iliacus Myositis and Bacteremia Caused by Non-typhoidal Salmonella in a Healthy Adult: A Case Report

**DOI:** 10.7759/cureus.52182

**Published:** 2024-01-12

**Authors:** Yu Miyazaki, Takuya Adachi

**Affiliations:** 1 Department of Infectious Diseases, Tokyo Metropolitan Toshima Hospital, Tokyo, JPN

**Keywords:** immunocompetent, extra-intestinal focal infection, abscess, bacteremia, iliacus myositis, non-typhoidal salmonella

## Abstract

Typically, non-typhoidal *Salmonella* (NTS) causes gastroenteritis; however, NTS can also lead to extraintestinal manifestations like bacteremia, meningitis, and abscess in vulnerable populations such as children, elderly, and immunocompromised individuals. Extraintestinal manifestations in an immunocompetent patient are uncommon. Here, we report a case of iliacus myositis and bacteremia caused by NTS in a healthy adult. A previously healthy 23-year-old Japanese woman presented to the emergency room due to a one-day history of vomiting, abdominal pain, and diarrhea. She had a fever, and her blood test showed leukocytosis. Computed tomography (CT) of the abdomen and pelvis revealed a thickening of the ascending colon. She was diagnosed with gastroenteritis and treated with antibiotics. Blood culture at that time was negative, but *Salmonella *serogroup 09 was detected in stool culture. Subsequently, her symptoms improved; however, on day 13, she returned to our hospital complaining of pain in her left thigh. CT scan of the abdomen and pelvis revealed no abscess or muscle inflammation, but due to persistent symptoms, the patient was admitted to the hospital, and antibiotics were initiated. *Salmonella* serogroup 09 was detected from the blood culture obtained at the time of admission. Magnetic resonance imaging of the hip joint and CT of the abdomen and pelvis after admission revealed inflammation in the left iliacus muscle and an abscess near the left ilium. The patient was treated successfully with antibiotics. This case highlights two findings. First, NTS can cause bacteremia even in a healthy adult with no risk factors for bacteremia. Second, NTS bacteremia can cause complications in an immunocompetent adult. This case implies that even in the case of NTS infection in otherwise healthy individuals, if there are signs of bacteremia or complications, it is essential to conduct blood culture and additional tests.

## Introduction

*Salmonella* species are Gram-negative rod bacterium belonging to the family *Enterobacteriaceae* [1]. It is classified into more than 2600 serotypes and approximately 50 of these serotypes have the potential to instigate infections in humans [2]. *Salmonella* species can be categorized into two main types: typhoidal* Salmonella* and non-typhoidal *Salmonella* (NTS) [3]. NTS is predominantly transmitted through contaminated food, water, and contact with animals such as reptiles [4]. Typically, it causes gastroenteritis; however, NTS can also lead to extraintestinal manifestations like bacteremia, meningitis, and abscess in vulnerable populations such as children, the elderly, and immunocompromised individuals [5]. Extraintestinal manifestations in NTS infection are uncommon in an immunocompetent individual, and only a few reports exist on the complications. We herein report a case of iliacus myositis and bacteremia caused by NTS in a healthy adult with no apparent exposure to contaminated food, water, or animals.

## Case presentation

A 23-year-old Japanese woman with no significant past medical history visited an emergency room due to a one-day history of vomiting, abdominal pain, and diarrhea. The patient denied any prior exposure to contaminated foods or contact with sick individuals or animals except for her two dogs. She had not traveled abroad for at least a year. The patient had a body temperature of 38.8℃, blood pressure of 122/70 mmHg, a pulse rate of 110 per minute, and a respiratory rate of 22 per minute. Her physical examination showed no apparent abnormalities. Laboratory tests included leukocytosis (19,400/μL) with 93.4% of neutrophils and an elevation of serum C-reactive protein (2.0 mg/dL) (Table 1). A computed tomography (CT) of the abdomen and pelvis revealed a thickening of the ascending colon. The patient was diagnosed with gastroenteritis and received ceftriaxone 2 g in the emergency room. Subsequently, the emergency physician prescribed fosfomycin 3000 mg daily for five days. Although blood culture (two sets, four bottles) did not reveal any growth, *Salmonella* serogroup 09 was detected in stool culture on day five. Subsequently, the symptoms gradually improved and the fever and nausea disappeared on day five. However, on day seven, the fever reappeared. The patient went to the clinic and was prescribed fosfomycin 3000 mg daily for five days. As the fever persisted, she presented to our outpatient department on day 11. Although her body temperature was 37.7℃, her blood test showed improved inflammatory markers. There were no abnormalities in the physical examination and the stool culture. She was placed under observation.

**Table 1:** Patient’s laboratory values. AST: aspartate aminotransferase; ALT: alanine aminotransferase; BUN: blood urea nitrogen; LDH: lactate dehydrogenase; CK: creatine kinase; BNP: brain natriuretic peptide.

	Laboratory values on day one	Laboratory values on day 13	Laboratory values on day 17	Normal laboratory value
WBC	19,400	11,000	8,800	3,300-8,600/μL
Neutrophils	93.4	74.9	69.2	37-80%
Hemoglobin	13.5	13.9	11.2	11.6-14.8 g/dL
Hematocrit	41.6	41.8	33.7	35.1-44.4%
Platelets	21.6	23.7	20.2	15.8-34.8 × 10^4^/μL
Total bilirubin	0.9	0.5	0.5	0.4-1.5 mg/dL
AST	20	88	90	13-30 U/L
ALT	12	84	88	7-23 U/L
LDH	196	471	333	124-222 U/L
CK	63	34	26	41-153 U/L
Total protein	7.0	7.6	5.4	6.6-8.1 g/dL
Albumin	4.6	4.2	2.5	4.1-5.1 g/dL
BUN	10.9	5.9	5.0	0.46-0.79 mg/dL
Creatinine	0.52	0.43	0.35	0.46-0.79 mg/dL
BNP	-	-	15.8	0.0-18.4 pg/mL
Troponin I	-	-	<0.01	0.00-0.03 ng/mL
C-reactive protein	2.0	2.9	13.8	0.00-0.14 mg/dL

**Figure 1:**
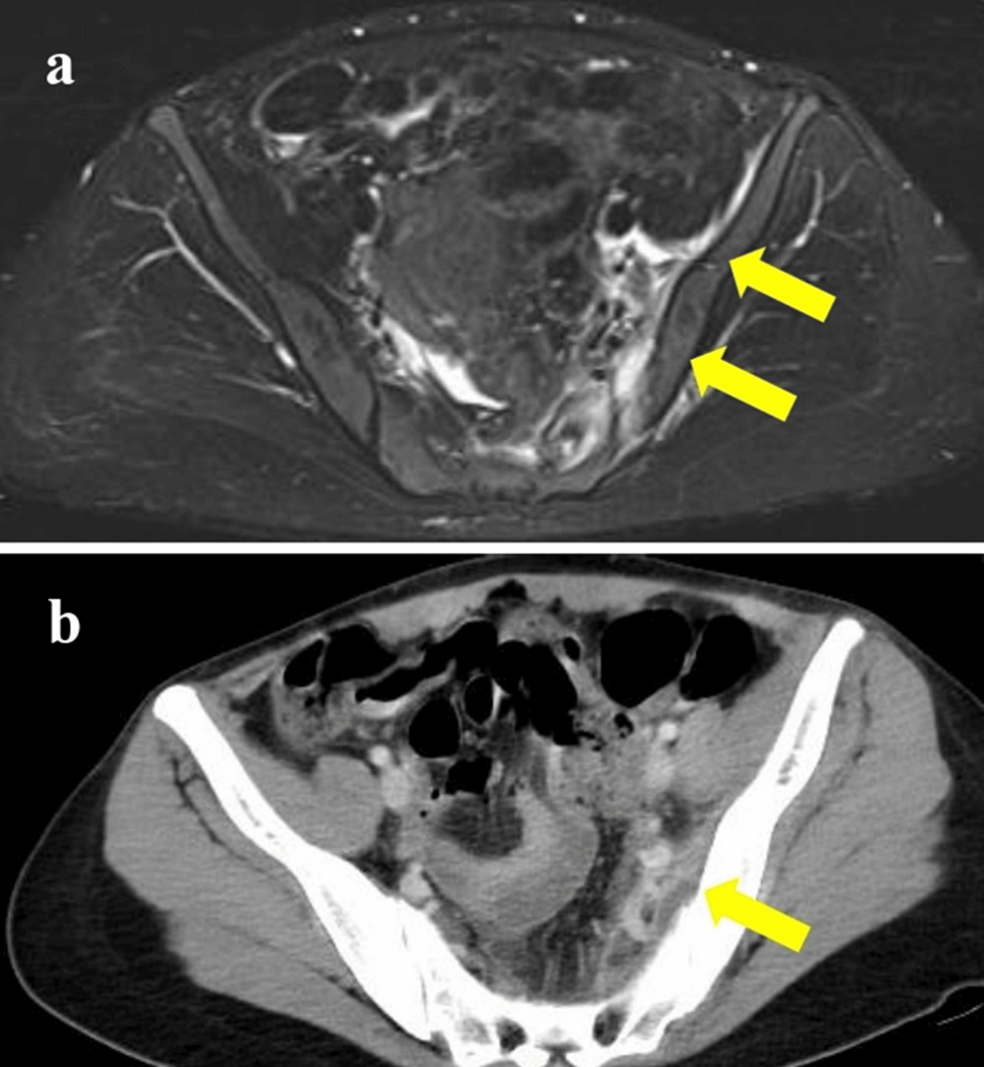
(A) Magnetic resonance imaging of the hip joint and (B) computed tomography of the pelvis. (A) Inflammation in the left iliacus muscle was demonstrated by an MRI (short tau inversion recovery) taken on day three of admission. (B) Abscess and fluid collection adjacent to the left ileum were demonstrated by CT taken on day four of admission.

On day 13, the patient developed left thigh pain and returned to our emergency room. Her vital signs were as follows: body temperature: 38.5℃; blood pressure: 124/75 mmHg; heart rate: 105 per minute; respiratory rate: 18 per minute; and oxygen saturation: 97% on ambient air. She experienced intense pain in the left thigh and was unable to walk. It was worsened by movement and extension of the left leg but she had an intact sensation in both lower extremities. Blood tests showed leukocytosis (11,000/μL) with 74.9% of neutrophils and an elevation of serum C-reactive protein (2.9 mg/dL) (Table 1). A contrast-enhanced CT scan of the abdomen and pelvis revealed no abscess or muscle inflammation, but due to persistent symptoms, the patient was admitted to the hospital and started on ceftriaxone 2 g daily. On the third day of admission, *Salmonella *serogroup 09 was detected from her blood culture obtained at the time of admission. Antimicrobial susceptibility testing showed that the organism was sensitive to levofloxacin (Table 2) and the treatment was switched to intravenous levofloxacin 500 mg daily. As the lower limb pain persisted after admission, an MRI scan of the hip joint was performed to evaluate the iliopsoas muscle, revealing inflammation in the left iliacus muscle (Figure 1A). Hypoxia appeared on the third day of admission, gradually worsening through the fourth day. The other vital signs remained stable on the fourth day; however, oxygen saturation decreased to 92% on ambient air. The laboratory test showed the following results: albumin level of 2.5 g/dL, brain natriuretic peptide (BNP) level of 15.8 pg/mL, and troponin I level of <0.01 ng/mL (Table 1). A contrast-enhanced CT scan revealed pleural effusion and an abscess near the left ilium (Figure 1B), but there was no abscess on the iliopsoas muscle. The echocardiogram showed a preserved ejection fraction and did not show any valvular vegetation or any evidence of endocarditis. We consulted with a cardiologist and pulmonologist. The cause of hypoxemia was determined to be pleural effusion due to hypoalbuminemia caused by NTS infection. As oxygen saturation was maintained at 95% during oxygen therapy at 1.5 L/min via nasal cannula, we continued antibiotics without performing thoracentesis. Consultation was sought regarding the drainage of the abscess; however, considering the small size of the abscess, it was determined that drainage was not necessary. During her hospitalization, her symptoms gradually improved. Oxygen therapy was discontinued on the fifth day of admission, and both pain and fever gradually subsided. She was subsequently switched to oral levofloxacin 500 mg daily on day eight of admission.

**Table 2:** Antimicrobial susceptibility profile of Salmonella serogroup 09. MIC: minimum inhibitory concentration.

Drug	Sensitivity	MIC
Ampicillin	Sensitive	<8
Piperacillin	Sensitive	<8
Ampicillin/sulbactam	Sensitive	<8
Piperacillin/tazobactam	Sensitive	<16
Cefotaxime	Sensitive	<1
Ceftriaxone	Sensitive	<1
Ceftazidime	Sensitive	<4
Cefcapene	Sensitive	0.5
Cefepime	Sensitive	<2
Flomoxef	Sensitive	<8
Cefoperazone/sulbactam	Sensitive	<16
Imipenem	Sensitive	<1
Meropenem	Sensitive	<1
Aztreonam	Sensitive	<4
Levofloxacin	Sensitive	<0.5
Sulfamethoxazole-trimethoprim	Sensitive	<2
Minocycline	Sensitive	<2
Fosfomycin	Sensitive	<4
Nalidixic acid	Sensitive	

Two weeks after admission, the patient was discharged home and prescribed oral levofloxacin 500 mg daily. CT scan was performed two weeks after discharge and showed significant regression in the abscess and pleural effusion. On her follow-up visit, the patient showed significant improvement, her pain had disappeared, and inflammatory markers had improved. She received antibiotics for 33 days and has remained stable since then.

## Discussion

Here, we report a case of iliacus myositis and bacteremia caused by NTS in a healthy adult in Japan. This case highlights two findings. First, NTS can cause bacteremia even in a healthy adult with no risk factors for bacteremia. Second, NTS bacteremia can cause complications in an immunocompetent adult.

First, NTS can cause bacteremia even in a healthy adult with no risk factors for bacteremia. It has been reported that 5% of enteric infections caused by NTS result in bacteremia [6]. The risk of bacteremia and complications includes factors such as old age, corticosteroid use, malignancy, diabetes, HIV, and other immunosuppressive drugs [6,7]. In terms of clinical symptoms, the risk factors for bacteremia include fever, abdominal pain, and diarrhea. In patients with bacteremia, there is a higher proportion of fever and diarrhea lasting for more than four days [8]. This case is rare because the patient was immunocompetent without any underlying diseases. Our patient presented with fever, abdominal pain, and diarrhea, which persisted for more than four days. We repeated a blood culture and despite the prior antibiotic treatment in this patient, her diagnosis was confirmed.

Second, NTS bacteremia can cause complications in an immunocompetent adult. There have been reports stating that 25% of patients aged 50 years and older with NTS bacteremia had complications, while no complications were observed in younger individuals [9]. Furthermore, there have been reports indicating that 1.7% of patients with NTS bacteremia experienced complications of psoas abscess [10], but there is no report of iliacus myositis. In this patient, although a contrast-enhanced CT scan at admission revealed no abscess or muscle inflammation, due to the persistence of symptoms after admission, an MRI examination was performed, which revealed left iliacus myositis. The CT scan after admission identified an abscess adjacent to the left ilium, which is the cause of left thigh pain. As possible causes of left iliacus myositis, both a hematogenous spread and direct expansion of a nearby site have been proposed. In this case, enteritis was distant from the iliac muscle, and the CT scan at admission did not indicate any signs of an abscess. Therefore, hematogenous spread associated with bacteremia was considered the cause of iliacus myositis. To the best of our knowledge, there have been no previous reports of healthy young patients developing NTS bacteremia and subsequently experiencing iliacus myositis and abscess adjacent to the ilium.

## Conclusions

Typically, NTS causes extraintestinal manifestations in immunocompromised patients. However, NTS can cause bacteremia and complications even in a healthy adult with no risk factors for bacteremia. This case implies that even in the case of NTS infection in otherwise healthy individuals, if there are signs of bacteremia or complications, it is essential to conduct blood culture and additional tests.
